# Relação entre Resposta Imune Inata do Receptor Toll-Like-4 (TLR-4) e o Processo Fisiopatológico da Cardiomiopatia da Obesidade

**DOI:** 10.36660/abc.20190788

**Published:** 2021-07-15

**Authors:** Pedro Henrique Rizzi Alves, Artur Junio Togneri Ferron, Mariane Róvero Costa, Fabiana Kurokawa Hasimoto, Cristina Schmitt Gregolin, Jéssica Leite Garcia, Dijon Henrique Salomé de Campos, Antônio Carlos Cicogna, Letícia de Mattei, Fernando Moreto, Silméia Garcia Zanati Bazan, Fabiane Valentini Francisqueti-Ferron, Camila Renata Corrêa

**Affiliations:** 1 Universidade Estadual Paulista Júlio de Mesquita FilhoFaculdade de MedicinaBotucatuSPBrasil Universidade Estadual Paulista Júlio de Mesquita Filho Câmpus de Botucatu Faculdade de Medicina , Botucatu , SP - Brasil; 2 Universidade Federal de Mato GrossoSinopMTBrasil Universidade Federal de Mato Grosso , Sinop , MT – Brasil

**Keywords:** Doenças Cardiovasculares/fisiopatologia, Obesidade, Inflamação, Citocinas, Adipócitos, Ácido Graxo Sintases, Avaliação Nutricional

## Abstract

**Fundamento:**

A obesidade é uma condição inflamatória crônica de baixo grau relacionada a distúrbios cardíacos. No entanto, o mecanismo responsável pela inflamação cardíaca relacionada à obesidade não é claro. O receptor do tipo toll 4 (TLR-4) pertence a um receptor da família das transmembranas, responsável pela resposta imune, cuja ativação estimula a produção de citocinas pró-inflamatórias.

**Objetivo:**

Testar se a ativação do receptor TLR-4 participa do processo de cardiomiopatia da obesidade, devido à produção de citocinas por meio da ativação do NF-ĸB.

**Métodos:**

Ratos Wistar machos foram randomizados em dois grupos: o grupo controle (C, n = 8 animais) que recebeu dieta padrão/água e o grupo obeso (OB, n = 8 animais) que foi alimentado com dieta rica em açúcar e gordura e água mais 25% de sacarose por 30 semanas. Análise nutricional: peso corporal, índice de adiposidade, alimentos, água e ingestão calórica. Análise de distúrbios relacionados à obesidade: glicose plasmática, ácido úrico e triglicerídeos, HOMA-IR, pressão arterial sistólica, TNF-α no tecido adiposo. A análise cardíaca incluiu: expressão das proteínas TLR-4 e NF-ĸB, níveis de TNF-α e IL-6. Comparação pelo teste t de *Student* não pareado ou teste de Mann-Whitney com um valor de p <0,05 como estatisticamente significativo.

**Resultados:**

O grupo OB apresentou obesidade, glicose elevada, triglicerídeos, ácido úrico, HOMA, pressão arterial sistólica e TNF-α no tecido adiposo. O grupo OB apresentou remodelação cardíaca e disfunção diastólica. A expressão de TLR-4 e NF-ĸB e os níveis de citocinas foram maiores em OB.

**Conclusão:**

Nossos achados concluem que, em uma condição obesogênica, a inflamação derivada da ativação do TLR-4 cardíaco pode ser um mecanismo capaz de levar à remodelação e disfunção cardíaca.

## Introdução

A obesidade, definida como um acúmulo excessivo de gordura corporal que pode prejudicar a saúde do indivíduo, ^1^ é atualmente considerada o distúrbio nutricional de maior importância em países desenvolvidos e subdesenvolvidos. ^2^ Estimativas mostram que pelo menos 18% da população adulta será obesa em 2025, uma condição preocupante uma vez que pode acarretar em diversas comorbidades, entre elas, as doenças cardiovasculares. ^3 , 4^

Uma das principais causas responsáveis pela atual epidemia de obesidade é o consumo excessivo de dietas hipercalóricas, ricas em gordura saturada e açúcares refinados, associadas ao estilo de vida sedentário. ^5 , 6^ Esse estilo de vida moderno leva a uma hipertrofia do tecido adiposo que desencadeia, inicialmente, um processo inflamatório local, que pode posteriormente afetar e comprometer a função de outros órgãos, como o coração. ^7^ Essa inflamação de origem metabólica tem como resultado a síntese de citocinas pró-inflamatórias através de diferentes vias, entre elas: lipólise do tecido adiposo, ^8^ hipertrofia dos adipócitos, ^9^ ácidos graxos da dieta ^10^ e lipopolissacarídeos intestinais (LPS) devido à disbiose. ^11^ Nesse contexto, os ácidos graxos livres e LPS são os principais componentes envolvidos na resposta inflamatória, agindo como Padrões Moleculares Associados a Danos ( *damage-associated molecular patterns –* DAMPs) e Padrões Moleculares Associados a Patógenos ( *pathogen-associated molecular patterns –* PAMPS), respectivamente, que são reconhecidos por alguns receptores, especialmente o receptor do tipo Toll 4 ( *Toll-like receptor 4 –* TLR-4). ^12^

O TLR-4 pertence a uma família de receptores geralmente expressos em células do sistema imune inato, como macrófagos, neutrófilos e linfócitos, e exerce um papel importante na detecção e reconhecimento de patógenos que levam à ativação das respostas imunes inata ^13^ e adquirida. ^14^ Esse receptor é responsável pela ativação do fator nuclear kappa B ( *nuclear factor kappa B –* NF-ĸB) com consequente síntese de citocinas pró-inflamatórias envolvidas na fisiopatologia de diversas doenças, inclusive cardiopatas. ^15^

No entanto, a literatura relata que o receptor TLR-4 não é expresso apenas em células do sistema imunológico. Os cardiomiócitos também expressam esse receptor e sua ativação, especialmente contra patógenos infecciosos cardíacos, contribui para o processo de disfunção miocárdica. ^16^ Embora o envolvimento do sistema imunológico no desenvolvimento da hipertrofia patológica cardíaca esteja bem estabelecido, ^14 , 19 , 20^ a participação das vias TLR- 4 no desenvolvimento e progressão da remodelação cardíaca relacionados à obesidade ainda não foi esclarecida. ^21^ Considerando a escassez de estudos a respeito desse assunto, o objetivo deste estudo foi testar a hipótese de que a ativação do receptor TLR-4 participa do processo de cardiomiopatia por obesidade, devido à produção de citocinas por meio da ativação do NF-ĸB.

## Materiais e Métodos

### Protocolo Experimental

Ratos Wistar machos (n = 16), com 21 dias de idade, foram obtidos na Universidade Estadual Paulista (UNESP). O tamanho da amostra foi calculado por meio do SigmaStat para Windows, versão 3.5 (Systat Software Inc., San Jose, CA, EUA), considerando as médias das diferenças esperadas de 2,0, desvio padrão esperado de 1,0, poder do teste de 90% e α de 0,05. Esse cálculo considerou o índice de adiposidade de estudos anteriores publicados por nosso grupo. ^22 - 24^ Os animais foram divididos aleatoriamente em dois grupos experimentais: o grupo controle (C, n = 8 animais) que recebeu dieta padrão/água e o grupo obeso (OB, n = 8 animais) que receberam dieta rica em açúcar e gordura ( *high sugar-fat diet –* HSF) e água mais 25% de sacarose por 30 semanas. O modelo de dieta é um modelo bem estabelecido para indução à obesidade publicado anteriormente por nosso grupo de pesquisa. ^25^ A dieta HSF continha farelo de soja, sorgo, casca de soja, dextrina, sacarose, frutose, banha, vitaminas e minerais, mais 25% de sacarose em água potável. A dieta controle continha farelo de soja, sorgo, casca de soja, dextrina, óleo de soja, vitaminas e minerais.

A ração e água foram oferecidas *ad libitum* e os animais mantidos em gaiolas individuais, em ambiente com temperatura (24 ± 2°C), umidade (55 ± 5%) e ciclo claro-escuro (12-12h) controlados. O protocolo do estudo (CEUA 1265/2018) foi aprovado pelo Comitê de Ética em Experimentação Animal da Faculdade de Medicina de Botucatu da UNESP, São Paulo, Brasil, e seguiu as recomendações do Guia para o Cuidado e Uso de Animais Experimentais. ^26^ Ao final das 30 semanas, os animais foram mantidos em jejum de 8h, eutanasiados e o material foi coletado para análise.

### Avaliação Nutricional

O consumo de ração e água foram avaliados diariamente. A ingestão calórica foi determinada multiplicando-se o valor energético de cada dieta (g × Kcal) pela ingestão alimentar diária. Para o grupo OB, a ingestão calórica também incluiu as calorias da água (0,25 × 4 × mL consumido). O peso corporal dos animais foi medido semanalmente. Após a eutanásia, a gordura visceral (GV), a gordura epididimal (GE) e a gordura retroperitoneal (GR) foram coletadas e utilizadas para calcular o índice de adiposidade (IA) pela seguinte fórmula: [(GV + GE + GR) / PESO ANIMAL] x100.

### Análise de distúrbios relacionados à obesidade

A glicose e os triglicerídeos plasmáticos (BioClin, Quibasa Química Básica Ltda., Belo Horizonte, Minas Gerais, Brasil) foram medidos pelo método colorimétrico-enzimático em sistema analisador enzimático automático (Chemistry Analyzer BS-200, MindrayMedical International Limited, Shenzhen, China). A insulina (EMD Millipore Corporation, Billerica, MA, EUA) foi analisada no plasma por ELISA usando um kit comercial. A leitura foi realizada por espectrofotômetro de microplacas Spectra Max 190 (Molecular Devices®, Sunnyvale, CA, EUA).

O *Homeostatic Model Assessment* (HOMA-IR), que permite avaliar a resistência à insulina, também foi calculado de acordo com a fórmula: insulina em jejum (μUI / mL) x glicose em jejum (mmol / L) / 22,5. ^27 , 28^

A avaliação da pressão arterial sistólica (PAS) foi avaliada em ratos conscientes por pletismografia com um NarcoBioSystems® Electro-Sphygmomanometer (International Biomedical, Austin, TX, EUA). Os animais foram aquecidos em uma caixa de madeira (50 x 40 cm) entre 38–40°C com calor gerado por duas lâmpadas incandescentes por 4–5 minutos para estimular a vasodilatação arterial. Após esse procedimento, um manguito com sensor de pulso pneumático foi fixado na cauda de cada animal. O manguito foi insuflado com pressão de 200 mmHg e posteriormente desinsuflado. Os valores da pressão arterial foram registrados em um polígrafo Gould RS 3200 (Gould Instrumental Valley View, Ohio, EUA). Uma média de três leituras de pressão foi registrada para cada animal.

Como a obesidade está associada a uma condição inflamatória no tecido adiposo, foram avaliados os níveis de TNF-α e IL-6. Tecido adiposo epididimal (400 mg) foi triturado com 2 ml de PBS (pH 7,4) e depois centrifugado a 3.000 rpm e 4°C durante 10 min. Usando os sobrenadantes, o TNF-α e a IL-6 foram medidos usando kits comerciais de ELISA (R&D Systems) de acordo com as instruções do fabricante. Os valores foram normalizados pelas quantidades de proteínas de cada amostra quantificada pelo método de Bradford ^29^ e os resultados expressos em picograma/g proteína (pg/g proteína). O tecido adiposo epididimal foi selecionado por apresentar padrão de inflamação semelhante ao encontrado na gordura visceral. ^30^

### Análise ecocardiográfica

A análise foi realizada nos animais vivos por ecocardiografia transtorácica, com sistema Vivid S6 equipado com transdutor ultrassônico multifrequencial de 5,0 a 11,5 MHz (General Electric Medical Systems, Tirat Carmel, Israel). Os animais foram levemente anestesiados por injeção intraperitoneal com uma mistura de cetamina (50 mg/kg) e xilazina (1 mg/kg) e colocados em decúbito esquerdo. As medidas estruturais das imagens cardíacas foram obtidas no modo unidimensional (modo M) guiadas pelas imagens no modo bidimensional com o transdutor na posição paraesternal, eixo menor. A avaliação do ventrículo esquerdo (VE) foi realizada com o cursor em modo M logo abaixo do plano da válvula mitral no nível dos músculos papilares. Todos os exames foram realizados pelo mesmo examinador e obtidos de acordo com o método principal recomendado pela Sociedade Americana de Ecocardiografia ( *American Society of Echocardiography* ). As imagens da aorta e do átrio esquerdo foram obtidas posicionando o curso do modo M para planejar o nível da válvula aórtica. Foram avaliadas as seguintes estruturas cardíacas: diâmetro diastólico do ventrículo esquerdo (DDVE); espessura da parede posterior do ventrículo esquerdo (EPP); espessura do septo interventricular (ESI); diâmetro da aorta (DA); átrio esquerdo (AE) e a espessura relativa da parede do VE (ERP). A função sistólica do VE foi avaliada pelo débito cardíaco e também pela frequência cardíaca (FC) por ser um modulador da função sistólica cardíaca. A função diastólica do VE foi avaliada pela velocidade de pico inicial do fluxo transmitral (E); tempo de desaceleração da onda E (tempo desacel.). O estudo foi complementado pela avaliação por Doppler tecidual diastólico precoce (E’) e tardio (A’) do anel mitral (velocidades médias aritméticas de deslocamento das paredes lateral e septal), e a relação das ondas (E/E’ e E’/A’).

### Análise do tecido cardíaco

#### Inflamação

Cem miligramas (100 mg) foram homogeneizados em tampão fosfato-salino ( *phosphatebuffered saline –* PBS) utilizando o sobrenadante. A quantificação de TNF-α e IL-6 foi realizada por ELISA usando um kit comercial (Linco Research Inc., R & D Systems, Millipore and B-Brigde International Inc.). A leitura foi realizada pelo espectrofotômetro de microplacas Spectra Max 190 (Molecular Devices®, Sunnyvale, CA, EUA). Os resultados foram corrigidos pela quantidade de proteína total de acordo com o método de Bradford.

## PCR em tempo real

O fragmento ventricular esquerdo congelado foi homogeneizado em TRIzol® para extração do ácido ribonucleico (RNA). Em seguida, o RNA foi submetido à transcrição reversa, conversão do RNA em ácido desoxirribonucleico complementar (cDNA), pela ação da enzima transcriptase reversa, utilizando o Sistema SuperScript II de Síntese de Primeira Cadeia para RT-PCR® Invitrogen, São Paulo, Brasil). O cDNA obtido foi utilizado na reação em cadeia da polimerase ( *polymerase chain reaction –* PCR) usando ensaios prontos (Applied Biosystems, CA, EUA) contendo o primer TaqMan MGB (FAM) e o primer específico para TLR-4 (Rn00569848_m1).

## Análise de Western Blot

Os fragmentos do coração foram homogeneizados em tampão de lise e centrifugados. O sobrenadante foi coletado e a concentração de proteína analisada pelo método de Bradford. ^29^ Após a quantificação, os extratos das proteínas cardíacas foram diluídos em solução tampão contendo Tris-HCl 50 mM (pH 6,8), 200 mM 2-Mercaptoetanol, dodecil sulfato de sódio ( *sodium dodecyl sulfate –* SDS) 2%, azul de bromofenol 0,1% e glicerol 10%. As diluições (50 ug) foram aquecidas e submetidas a eletroforese em gel de SDS/gel de eletroforese de poliacrilamida ( *sodium dodecyl sulfate/polyacrylamide gel electrophoresis –* SDS-PAGE) em géis de poliacrilamida a 10%. Após a eletroforese, as proteínas foram eletrotransferidas para membranas de nitrocelulose (Bio-Rad Biosciences; NJ, EUA). Os sítios de ligação não específicos do anticorpo primário para a membrana foram bloqueados por incubação com solução de leite em pó desnatado a 0,5%, dissolvido em tampão TBS-T pH 7,4. A membrana foi então lavada três vezes em solução basal e incubada durante a noite com o anticorpo primário específico para TLR-4 (sc293072), NF-ĸB total (sc8008), NF-ĸB fosforilado (ser536) (sc33020). A β-actina foi usada como controle interno (sc47778). Após a incubação, as membranas foram lavadas e incubadas com os respectivos anticorpos secundários. Por fim, a imunodetecção foi realizada pelo método de quimioluminescência, de acordo com as instruções do fabricante (ECL SuperSignal® West Pico Chemiluminescent Substrate – Thermo Scientific, Rockford, IL, USA, 34080), e analisada por meio de um densitômetro (densitômetro de imagem calibrado GS-710, Laboratório Bio-Rad, CA, EUA).

## Análise estatística

Os dados foram submetidos ao teste de normalidade Kolmogorov-Smirnov. As variáveis paramétricas foram comparadas pelo teste *t* de *Student* não pareado e os resultados expressos como média ± desvio padrão. As variáveis não paramétricas foram comparadas pelo teste de Mann-Whitney e os resultados expressos em mediana (intervalo interquartil 25-75). As análises estatísticas foram realizadas usando o Sigma Stat para Windows, versão 3.5 (Systat Software Inc., San Jose, CA, EUA). Um valor de p <0,05 foi considerado estatisticamente significativo.

## Resultados

A Tabela 1 mostra os parâmetros nutricionais. Os animais OB consumiram menos ração, mas mais água em comparação ao grupo controle, refletindo em ingestão calórica semelhante. O grupo OB também apresentou obesidade caracterizada por aumento do peso corporal e índice de adiposidade.

**Tabela 1:** – Parâmetros nutricionais

Características	Grupos						
	Controle (n=8)			OB (n=8)			Valor de p
Peso corporal final (g)	493	±	50,8	583	±	75,9	0,001*
Índice de adiposidade (%)	4,79	±	0,73	8,68	±	1,76	0,0004*
Consumo de ração (g/dia)	23,92	±	2,37	13,0	±	2,20	0,0001*
Consumo de água (ml/dia)	34,6	±	5,08	41,6	±	3,47	0,001*
Ingestão calórica (Kcal/dia)	85,9	±	8,52	98,3	±	8,35	0,07

OB: grupo obeso. Dados expressos como média ± desvio padrão. Comparação pelo teste t de Student não pareado. *indica p <0,05.

Em relação aos distúrbios relacionados à obesidade, o grupo OB apresentou glicose, triglicérides e ácido úrico mais elevados, resistência à insulina, aumento da pressão arterial sistólica e inflamação do tecido adiposo, com níveis elevados de TNF-α e IL-6 em relação ao grupo C ( Tabela 2 ).

**Tabela 2:** – Distúrbios relacionados à obesidade

Características	Grupos						
	Controle (n=8)			OB (n=8)			Valor de p
Glicose (mg/dL)	75,7	±	2,02	105	±	17,9	0,02*
Triglicerídeos (mg/dL)	63,5	±	18,6	104	±	25,4	0,003*
Ácido úrico (mg/dL)	0,44	±	0,09	0,62	±	0,20	0,04*
HOMA-IR	5,90	±	2,32	30,9	±	17,0	0,004*
Pressão Arterial Sistólica (mmHg)	121	±	5,77	128	±	6,48	0,03*
Tecido adiposo TNF-α (pg/g proteína)	52,7 (46,6 – 62,5)			152 (117 – 219)			0,001*
Tecido adiposo IL-6 (pg/g proteína)	13,0 ± 9,2			94,5 ± 33,3			< 0,001*

OB: grupo obeso; HOMA-IR: Homeostatic Model Assessment. Dados expressos em média ± desvio padrão ou mediana (intervalo interquartil). Comparação pelo teste t de Student não pareado ou Mann-Whitney. *indica p<0,05.

A análise ecocardiográfica é apresentada na Tabela 3 . Ao final de 30 semanas, o grupo OB apresentou remodelação cardíaca, caracterizada por redução do diâmetro diastólico do ventrículo esquerdo (DDVE) e aumento da espessura da parede posterior do ventrículo esquerdo (EPP), espessura do septo interventricular (ESI), átrio esquerdo (AE) e diâmetro da aorta (DA). Além disso, os animais obesos apresentaram disfunção diastólica, representada por alterações na onda E, tempo de desaceleração da onda E e E’/A’.

**Tabela 3:** – Análise ecocardiográfica

Características	Grupos						
	Controle			OB			Valor de p
DDVE (mm)	7,20	±	0,20	6,70	±	0,56	0,031*
DDVE/PC	14,7	±	1,6	11,9	±	1,3	0,002*
EPP (mm)	3,03	±	0,31	3,35	±	0,21	0,033*
ESI (mm)	3,35	±	0,21	3,57	±	0,19	0,045*
ERP	0,45	±	0,05	0,51	±	0,09	0,098
DA (mm)	3,73	±	0,12	3,99	±	0,13	0,001*
AE (mm)	4,75	±	0,12	5,17	±	0,38	0,011*
FC (bpm)	234	±	39	295	±	26	0,002*
Débito cardíaco	0,91	±	0,01	0,88	±	0,06	0,142
Tempo de desaceleração (ms)	47,2	±	3,0	50,6	±	2,7	0,035*
Onda E	68,3	±	5,2	73,1	±	3,3	0,046*
Onda A	40,7	±	3,7	45,7	±	6,1	0,062
Razão E/A	1,68	±	0,08	1,61	±	0,016	0,327
Razão média E’/A’	1,53	±	0,14	1,26	±	0,25	0,018*
Razão média E/ E’	12	±	1,3	13,5	±	1,86	0,094

OB: grupo obeso; DDVE: diâmetro diastólico do ventrículo esquerdo; PC: peso corporal; EPP: espessura da parede posterior do ventrículo esquerdo; ESI: espessura do septo interventricular; ERP: espessura relativa da parede do ventrículo esquerdo; DA: diâmetro da aorta; AE: átrio esquerdo; FC: frequência cardíaca. Velocidade de pico inicial do fluxo transmitral (onda E); tempo de desaceleração: tempo de desaceleração da onda E. Doppler diastólico precoce (E’) e tardio (A’) do anel mitral (velocidades de deslocamento médias aritméticas das paredes lateral e septal) e a razão (E/E’ e E’/A’). Dados expressos como média ± desvio padrão. Comparação pelo teste t de Student não pareado. *indica p <0,05.

**Figura 1:**
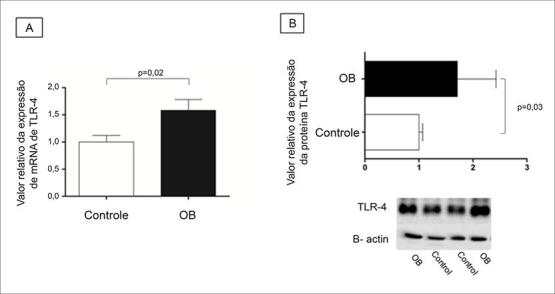
-Expressão de TLR-4 no tecido cardíaco. (A) Expressão do gene por PCR em tempo real; (B) Expressão de proteína Western Blot. Resultados expressos em média ± desvio padrão. Comparação pelo teste t de Student não pareado. *indica p <0,05; n = 8 animais/grupo. OB: Grupo obeso.

Em relação à expressão do gene TLR-4 e da proteína no tecido cardíaco, é possível verificar que ambos estavam aumentados no grupo OB ( Figura 1 ).

**Figura 2:**
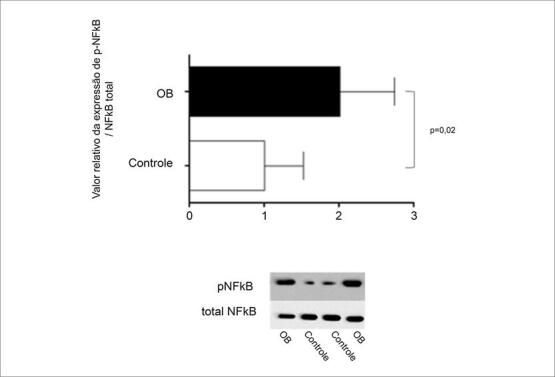
- Expressão da fosfoproteína e NF-ĸB total no tecido cardíaco. Resultados expressos em média ± desvio padrão. Comparação pelo teste t de Student não pareado. *indica p <0,05; n = 8 animais/grupo.

**Figura 3:**
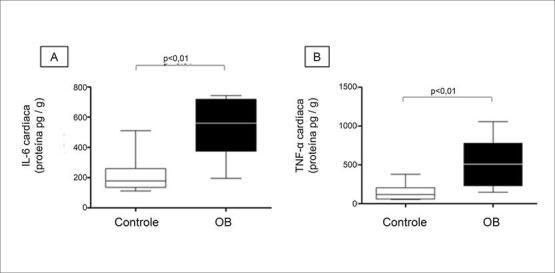
– Citocinas cardíacas no tecido cardíaco. (A) IL-6 (proteína pg/g); (B) TNF-α. Resultados expressos em mediana e intervalo interquartil. Comparação pelo teste de Mann-Whitney. *indica p <0,05; n = 8 animais/grupo.

A fosforilação do NF-ĸB no tecido cardíaco também foi maior no grupo OB ( Figura 2 ), resultando em aumento das citocinas, uma vez que esse grupo apresentou níveis mais elevados de TNF-α e IL-6 em relação ao grupo C ( Figura 3 ).

## Discussão

Este estudo hipotetizou que a ativação do receptor TLR-4 participa da doença cardíaca relacionada à obesidade por desencadear a produção de citocinas via NF-ĸB. Para induzir a obesidade e os distúrbios relacionados ao excesso de gordura corporal, os animais do grupo OB foram submetidos a uma dieta rica em açúcar e gordura por 30 semanas. Ao final do período experimental, os resultados mostraram que esses animais apresentavam maior índice de adiposidade assim com diversos distúrbios como hiperglicemia, aumento do ácido úrico, resistência à insulina, hipertrigliceridemia, elevação da PAS, e aumento dos níveis de TNF-α e IL-6 em coração e tecido adiposo, confirmando a eficácia do modelo de dieta utilizado. ^22^

A coexistência de distúrbios relacionados à obesidade — como resistência a insulina, diabetes e dislipidemia — associada à disfunção do tecido adiposo, caracterizada por desequilíbrio das adipocinas, promove respostas desadaptativas no coração, como hipertrofia de miócitos, disfunção contrátil e remodelação cardíaca, que contribuem para o desenvolvimento de ambos e para a progressão da insuficiência cardíaca crônica. ^31^ Essa condição foi confirmada neste estudo, uma vez que a avaliação ecocardiográfica mostrou remodelação cardíaca e disfunção diastólica no grupo OB. É importante ressaltar que as características concêntricas da remodelação cardíaca apresentadas no grupo OB são decorrentes do aumento da sobrecarga funcional e da manutenção dessa condição, levando a uma disfunção diastólica cardíaca. A literatura apresenta resultados semelhantes relacionados ao modelo de obesidade induzida por dieta. ^34^

A literatura relata que a remodelação cardíaca também pode ser liderada por altas concentrações de citocinas pró-inflamatórias, como o TNF-α e a IL-6. ^35 , 36^ O TNF-α tem sido implicado no desenvolvimento de disfunção ventricular esquerda, remodelação ventricular esquerda, aumento da apoptose de miócitos cardíacos e sua ação direta é exercida por receptores de TNF-α, que são expressos por quase todas as células nucleadas. Níveis elevados de IL-6 também podem induzir a hipertrofia do miócito e a disfunção miocárdica. ^37^ É importante enfatizar que essas citocinas podem ser produzidas em resposta à ativação da via TLR-4, e o L-6 parece também ser liberado em resposta direta ao TNF-α, ^35^ exacerbando as alterações cardíacas devido ao quadro inflamatório. Portanto, nossos achados confirmam as evidências a respeito da cardiopatia e inflamação da obesidade, uma vez que os níveis cardíacos de TNF-α e IL-6 foram maiores no grupo OB. ^18 , 37^

O envolvimento entre as citocinas pró-inflamatórias e o remodelamento cardíaco relacionado à obesidade induzida pela dieta pode ser atribuído a várias causas. ^18 , 38^ Assim, o objetivo deste estudo foi avaliar a ativação do TLR-4 cardíaco como responsável pelo desencadeamento do processo inflamatório. Observou-se que o grupo OB apresentou maior expressão do gene e da proteína TLR-4 juntamente com aumento da fosforilação do NF-ĸB, confirmando a ativação dessa via como mediadora da inflamação. A literatura relata que esse receptor pode ser ativado por lipopolissacarídeos (LPS) de bactérias gram-negativas, assim como por ácidos graxos. ^39^ Na obesidade, a lipólise do tecido adiposo representa uma importante fonte de ácidos graxos livres, capazes de ativar a via inflamatória. Esse processo catabólico pode ocorrer devido à resistência à insulina, uma vez que o tecido adiposo torna-se resistente ao efeito antilipolítico do hormônio. ^40 , 41^ Junto com a resistência à insulina, níveis elevados de TNF-α e IL-6 também são capazes de induzir lipólise no tecido adiposo. ^42 , 43^ Essas duas condições lipolíticas foram apresentadas pelo grupo OB. Além disso, em associação a esses mecanismos descritos acima, alguns pesquisadores relatam que o padrão alimentar ocidental está associado a alterações na microbiota intestinal, tornando esse órgão mais permeável, permitindo a translocação de bactérias patogênicas para a circulação. ^42^ O LPS bacteriano pode ser reconhecido por TLR-4, desencadeando a ativação do NF-ĸB com consequente síntese de citocinas. Embora esse mecanismo não tenha sido avaliado neste experimento, essa relação já está bem estabelecida. ^43 - 45^ Além disso, a composição da dieta utilizada neste estudo também está diretamente relacionada à ativação do receptor TLR-4, uma vez que os ácidos graxos saturados oferecidos no dieta tem LPS estrutural semelhante ao bacteriano, podendo também ser reconhecida, levando assim a um processo inflamatório pela via TLR-4. ^46 , 47^

## Conclusão

Em suma, todos esses mecanismos podem ter sido ativados de forma sinérgica, potencializando a produção de citocinas que desempenham papel fundamental no desenvolvimento das cardiomiopatias. Assim, este estudo demonstrou que a resposta imune inata por meio da ativação do receptor TLR-4 é um dos mecanismos que pode contribuir para o surgimento do processo inflamatório miocárdico na obesidade. Portanto, como não encontramos na literatura estudos que mostrassem interação entre essa via inflamatória, doença cardíaca e obesidade, nossos achados concluem que, em uma condição obesogênica, a inflamação derivada da ativação do TLR-4 cardíaco é um novo mecanismo que pode levar à remodelação e disfunção cardíaca.

## References

[1] . World Health Organization . Obesity and overweight [ Internet ]. Geneva : WHO ; 2011 [ acesso 5 de maio de 2016 ]. Disponível em: http://www.who.int//mediacentre/factsheets/fs311/en . 2016

[2] . Francischi RPP , Pereira LO , Freitas CS , Klopfer M , Santos RC , Vieira P , et al . Obesidade: atualização sobre sua etiologia, morbidade e tratamento . Rev Nutr . 2000 ; 13 ( 1 ): 17 - 28 .

[3] . Bhat ZF , Morton JD , Mason S , Bekhit AEDA , Bhat HF . Obesity and neurological disorders: dietary perspective of a global menace . Crit Rev Food Sci Nutr . 2019 ; 59 ( 8 ): 1294 - 310 . 10.1080/10408398.2017.1404442 29257910

[4] . Mandviwala T , Khalid U , Deswal A . Obesity and cardiovascular disease : a risk factor or a risk marker ? Curr Atheroscler Rep . 2016 ; 18 ( 5 ): 21 . 10.1007/s11883-016-0575-4 26973130

[5] . Johnson AR , Wilkerson MD , Sampey BP , Troester MA , Hayes DN , Makowski L . Cafeteria diet-induced obesity causes oxidative damage in white adipose . Biochem Biophys Res Commun . 2016 ; 473 ( 2 ): 545 - 50 . 10.1016/j.bbrc.2016.03.113 PMC4862365 27033600

[6] . Bray GA , Nielsen SJ , Popkin BM . Consumption of high-fructose corn syrup in beverages may play a role in the epidemic of obesity . Am J Clin Nutr . 2004 ; 79 ( 4 ): 537 - 43 . 10.1093/ajcn/79.4.537 15051594

[7] . Manna P , Jain SK . Obesity, oxidative stress, adipose tissue dysfunction, and the associated health risks: causes and therapeutic strategies . Metab Syndr Relat Disord . 2015 ; 13 ( 10 ): 423 - 44 . 10.1089/met.2015.0095 PMC4808277 26569333

[8] . Blüher M . Adipose tissue dysfunction in obesity . Exp Clin Endocrinol Diabetes . 2009 ; 117 ( 6 ): 241 - 50 . 10.1055/s-0029-1192044 19358089

[9] . Skurk T , Alberti-Huber C , Herder C , Hauner H . Relationship between adipocyte size and adipokine expression and secretion . J Clin Endocrinol Metab . 2007 ; 92 ( 3 ): 1023 - 33 . 10.1210/jc.2006-1055 17164304

[10] . Kramer B , França LM , Zhang Y , Paes AM de A , Martin Gerdes A , Carrillo-Sepulveda MA . Western diet triggers toll-like receptor 4 signaling-induced endothelial dysfunction in female wistar rats . Am J Physiol Heart Circ Physiol . 2018 ; 315 ( 6 ): H1735 - 47 . 10.1152/ajpheart.00218.2018 30265151

[11] . Del Bas JM , Guirro M , Boqué N , Cereto A , Ras R , Crescenti A , et al . Alterations in gut microbiota associated with a cafeteria diet and the physiological consequences in the host . Int J Obes . 2018 ; 42 ( 4 ): 746 - 54 . 10.1038/ijo.2017.284 29167556

[12] . Huang S , Rutkowsky JM , Snodgrass RG , Ono-Moore KD , Schneider DA , Newman JW , et al . Saturated fatty acids activate TLR-mediated proinflammatory signaling pathways . J Lipid Res . 2012 ; 53 ( 9 ): 2002 - 13 . 10.1194/jlr.D029546 PMC3413240 22766885

[13] . Ferraz EG , Silveira BBB , Sarmento VA , Santos JN . Toll-Like Receptors : regulation of the immune responses . Rev Gaúcha Odontol . 2011 ; 59 ( 3 ): 483 - 90 .

[14] . Yuan X , Deng Y , Guo X , Shang J , Zhu D , Liu H . Atorvastatin attenuates myocardial remodeling induced by chronic intermittent hypoxia in rats: partly involvement of TLR-4/MYD88 pathway . Biochem Biophys Res Commun . 2014 ; 446 ( 1 ): 292 - 7 . 10.1016/j.bbrc.2014.02.091 24582748

[15] . Frantz S , Kobzik L , Kim Y , Fukazawa R , Medzhitov R , Lee RT , et al . Toll4 (TLR4) expression in cardiac myocytes in normal and failing myocardium . 1999 ; 104 ( 3 ): 271 - 80 . 10.1172/JCI6709 PMC408420 10430608

[16] . Liu T , Zhang M , Niu H , Liu J , Ruilian M , Wang Y , et al . Astragalus polysaccharide from Astragalus Melittin ameliorates inflammation via suppressing the activation of TLR-4/NF-κB p65 signal pathway and protects mice from CVB3-induced virus myocarditis . Int J Biol Macromol . 2019 Apr 1 ; 126 : 179 - 86 . 10.1016/j.ijbiomac.2018.12.207 30586589

[17] . Liu F , Wen Y , Kang J , Wei C , Wang M , Zheng Z , et al . Regulation of TLR4 expression mediates the attenuating effect of erythropoietin on inflammation and myocardial fibrosis in rat heart . Int J Mol Med . 2018 ; 42 ( 3 ): 1436 - 44 . 10.3892/ijmm.2018.3707 PMC6089778 29845292

[18] . Zhang WB , Zhang HY , Zhang Q , Jiao FZ , Zhang H , Wang LW , et al . Glutamine ameliorates lipopolysaccharide-induced cardiac dysfunction by regulating the toll-like receptor 4/mitogen-activated protein kinase/nuclear factor-κB signaling pathway . Exp Ther Med . 2017 ; 14 ( 6 ): 5825 - 32 . 10.3892/etm.2017.5324 PMC5740782 29285127

[19] . Fuster JJ , Ouchi N , Gokce N , Walsh K . Obesity-induced changes in adipose tissue microenvironment and their impact on cardiovascular disease . Circ Res . 2016 ; 118 ( 11 ): 1786 - 808 . 10.1161/CIRCRESAHA.115.306885 PMC4887147 27230642

[20] . Krejci J , Mlejnek D , Sochorova D , Nemec P . Inflammatory cardiomyopathy : a current view on the pathophysiology , diagnosis , and treatment . Biomed Res Int . 2016 ; 2016 ( 4087632 ). 10.1155/2016/4087632 PMC4921131 27382566

[21] . Li F , Zhang H , Yang L , Yong H , Qin Q , Tan M , et al . NLRP3 deficiency accelerates pressure overload-induced cardiac remodeling via increased TLR4 expression . J Mol Med . 2018 ; 96 ( 11 ): 1189 - 202 . 10.1007/s00109-018-1691-0 30159587

[22] . Francisqueti FV , Ferron AJT , Hasimoto FK , Alves PHR , Garcia JL , Santos KC , et al . Gamma oryzanol treats obesity- induced kidney injuries by modulating the adiponectin receptor 2 / PPAR- α axis . Oxid Med Cell Longev . 2018 Sep 05 ; 2 : 1 - 9 . 10.1155/2018/1278392 PMC6151244 30271526

[23] . Costa MR , Garcia JL , Silva CCV , Ferron AJT , Francisqueti-Ferron FV , Hasimoto FK , et al . Lycopene modulates pathophysiological processes of non-alcoholic fatty liver disease in obese rats . Antioxidants . 2019 ; 8 ( 8 ): 276 . 10.3390/antiox8080276 PMC6720442 31387231

[24] . Ferron AJT , Aldini G , Francisqueti-Ferron FV , Silva CCVA , Bazan SGZ , Garcia JL , et al . Protective effect of tomato-oleoresin supplementation on oxidative injury recoveries cardiac function by improving β-adrenergic response in a diet-obesity induced model . Antioxidants . 2019 ; 8 ( 9 ): 368 . 10.3390/antiox8090368 PMC6770924 31480719

[25] . Francisqueti FV , Minatel IO , Ferron AJT , Bazan SGZ , Silva VS , Garcia JL , et al . Effect of gamma-oryzanol as therapeutic agent to prevent cardiorenal metabolic syndrome in animals submitted to high sugar-fat diet . Nutrients . 2017 ; 9 ( 12 ): 1299 . 10.3390/nu9121299 PMC5748750 29186059

[26] . Olfert ED , Cross BM , McWilliam AA . Guide to the care and use of experimental animals . Vol. 1 . Ottawa : Canadian Council on Animal Care ; 1993 .

[27] . Matthews DR , Hosker JP , Rudenski AS , Naylor BA , Treacher DF , Turner RC . Homeostasis model assessment: insulin resistance and beta-cell function from fasting plasma glucose and insulin concentrations in man . Diabetologia . 1985 ; 28 ( 7 ): 412 - 9 . 10.1007/BF00280883 3899825

[28] . Ferron A , Jacobsen BB , Sant’Ana P , Campos D , Tomasi L , Luzivotto R , et al . Cardiac dysfunction induced by obesity is not related to β -adrenergic system impairment at the receptor-signalling pathway . PLoS One . 2015 ; 10 ( 9 ): e0138605 . 10.1371/journal.pone.0138605 PMC4577087 26390297

[29] . Bradford MM . A rapid and sensitive method for the quantitation of microgram quantities of protein utilizing the principle of protein-dye binding . Anal Biochem . 1976 ; 72 ( 1-2 ): 248 - 54 . 10.1016/0003-2697(76)90527-3 942051

[30] . Luvizotto RAM , Nascimento AF , Imaizumi E , Pierine DT , Conde SJ , Correa CR , et al . Lycopene supplementation modulates plasma concentrations and epididymal adipose tissue mRNA of leptin , resistin and IL-6 in diet-induced obese rats . Br J Nutr . 2013 ; 110 ( 10 ): 1803 - 9 . 10.1017/S0007114513001256 23632237

[31] . Jacob PS , Fujii TMM , Yamada M , Borges MC , Pantaleão LC , Borelli P , et al . Isocaloric intake of a high-fat diet promotes insulin resistance and inflammation in Wistar rats . Cell Biochem Funct . 2013 ; 31 ( 3 ): 244 - 53 . 10.1002/cbf.2894 23008133

[32] . Lawler HM , Underkofler CM , Kern PA , Erickson C , Bredbeck B , Rasouli N . Adipose tissue hypoxia, inflammation and fibrosis in obese insulin sensitive and obese insulin resistant subjects . J Clin Endocrinol Metab . 2016 ; 101 ( 4 ): 1422 - 8 . 10.1210/jc.2015-4125 PMC4880157 26871994

[33] . Moreno-Fernández S , Garcés-Rimón M , Vera G , Astier J , Landrier JF , Miguel M . High fat/high glucose diet induces metabolic syndrome in an experimental rat model . Nutrients . 2018 ; 10 ( 10 ): 1502 . 10.3390/nu10101502 PMC6213024 30322196

[34] . Ferron AJT , Francisqueti FV , Minatel IO , Silva CCVA , Bazan SGZ , Kitawara KAH , et al . Association between cardiac remodeling and metabolic alteration in an experimental model of obesity induced by Western Diet . Nutrients . 2018 ; 10 ( 11 ): 1675 . 10.3390/nu10111675 PMC6266980 30400581

[35] . Hedayat M , Mahmoudi MJ , Rose NR , Rezaei N . Proinflammatory cytokines in heart failure: double-edged swords . Heart Fail Rev . 2010 ; 15 ( 6 ): 543 - 62 . 10.1007/s10741-010-9168-4 20405319

[36] . Nishida K , Otsu K . Inflammation and metabolic cardiomyopathy . Cardiovasc Res . 2017 ; 113 ( 4 ) 389 - 98 . 10.1093/cvr/cvx012 28395010

[37] . Anker SD , Haehling S . Inflammatory mediators in chronic heart failure: an overview . Heart . 2004 ; 90 ( 4 ): 464 - 70 . 10.1136/hrt.2002.007005 PMC1768165 15020532

[38] . Mocan M , Mocan Hognogi LD , Anton FP , Chiorescu RM , Goidescu CM , Stoia MA , et al . Biomarkers of inflammation in left ventricular diastolic dysfunction . Dis Markers . 2019 Jun 2 ; 2019 : 7583690 . 10.1155/2019/7583690 PMC6589287 31275453

[39] . Sopasakis VR , Sandstedt J , Johansson M , Lundqvist A , Bergström G , Jeppsson A , et al . Toll-like receptor-mediated inflammation markers are strongly induced in heart tissue in patients with cardiac disease under both ischemic and non-ischemic conditions . Int J Cardiol . 2019 Oct 15 ; 293 : 238 - 47 . 10.1016/j.ijcard.2019.06.033 31230935

[40] . Francisqueti FV , Nascimento AF , Minatel IO , Dias MC , Luvizotto RDAM , Berchieri-Ronchi C , et al . Metabolic syndrome and inflammation in adipose tissue occur at different times in animals submitted to a high-sugar/fat diet . J Nutr Sci . 2017 Aug 21 ; 6 : e41 . 10.1017/jns.2017.42 PMC5672321 29152245

[41] . Ruiz-núñez B , Dijck-Brouwer DAJ , Muskiet FAJ . The relation of saturated fatty acids with low-grade inflammation and cardiovascular disease . J Nutr Biochem . 2016 Oct ; 36 : 1 - 20 . 10.1016/j.jnutbio.2015.12.007 27692243

[42] . Langin D , Arner P . Importance of TNFα and neutral lipases in human adipose tissue lipolysis . Trends Endocrinol Metab . 2006 ; 17 ( 8 ): 314 - 20 . 10.1016/j.tem.2006.08.003 16938460

[43] . Hall G , Steensberg A , Sacchetti M , Fischer C , Keller C , Schjerling P , et al . Interleukin-6 stimulates lipolysis and fat oxidation in humans . J Clin Endocrinol Metab . 2003 ; 88 ( 7 ): 3005 - 10 . 10.1210/jc.2002-021687 12843134

[44] . Rinninella E , Cintoni M , Raoul P , Lopetuso LR , Scaldaferri F , Pulcini G , et al . Food components and dietary habits: keys for a healthy gut microbiota composition . Nutrients . 2019 ; 11 ( 10 ): 2393 . 10.3390/nu11102393 PMC6835969 31591348

[45] . Ubanako P , Xelwa N , Ntwasa M . LPS induces inflammatory chemokines via TLR-4 signalling and enhances the Warburg Effect in THP-1 cells . PLoS One . 2019 ; 14 ( 9 ): e1222614 . 10.1371/journal.pone.0222614 PMC6764657 31560702

[46] . Li P , Wu YH , Zhu YT , Li MX , Pei HH . Requirement of Rab21 in LPS-induced TLR4 signaling and pro-inflammatory responses in macrophages and monocytes . Biochem Biophys Res Commun . 2019 ; 508 ( 1 ): 169 - 76 . 10.1016/j.bbrc.2018.11.074 30471852

[47] . Fonceca AM , Zosky GR , Bozanich EM , Sutanto EN , Kicic A , McNamara PS , et al . Accumulation mode particles and LPS exposure induce TLR-4 dependent and independent inflammatory responses in the lung . Respir Res . 2018 ; 19 ( 15 ): 1 - 10 . 10.1186/s12931-017-0701-z PMC5778683 29357863

