# Supplementary material for: Relação entre Resposta Imune Inata do Receptor Toll-Like-4 (TLR-4) e o Processo Fisiopatológico da Cardiomiopatia da Obesidade
Source: Arq Bras Cardiol. 2021 Jul 15;117(1):91–9. [Article in Portuguese] doi: 10.36660/abc.20190788 (PMC8294729; doi:10.36660/abc.20190788)
Supplement: Translation

# Translation: Relationship between Innate Immune Response Toll-Like Receptor 4 (TLR-4) and the Pathophysiological Process of Obesity Cardiomyopathy

**Authors:** Pedro Henrique Rizzi Alves, Artur Junio Togneri Ferron, Mariane Róvero Costa, Fabiana Kurokawa Hasimoto, Cristina Schmitt Gregolin, Jéssica Leite Garcia, Dijon Henrique Salomé de Campos, Antônio Carlos Cicogna, Letícia de Mattei, Fernando Moreto, Silméia Garcia Zanati Bazan, Fabiane Valentini Francisqueti-Ferron, Camila Renata Corrêa

**Affiliations:** 1 Brazil Universidade Estadual Paulista Júlio de Mesquita Filho Câmpus de Botucatu Faculdade de Medicina, Botucatu, SP - Brazil; 2 Brazil Universidade Federal de Mato Grosso, Sinop, MT – Brazil

**Keywords:** Cardiovascular Diseases/physiopathology, Obesity, Inflammation, Cytokines, Adipocytes, Fatty Acid synthases, Nutrional Assessment

## Abstract

**Background:**

Obesity is a chronic low-grade inflammation condition related to cardiac disorders. However, the mechanism responsible for obesity-related cardiac inflammation is unclear. The toll-like receptor 4 (TLR-4) belongs to a receptor of the transmembrane family responsible for the immune response whose activation stimulates the production of proinflammatory cytokines.

**Objective:**

To test whether the activation of the TLR-4 receptor participates in the obesity cardiomyopathy process, due to cytokine production through NF-ĸB activation.

**Methods:**

Male Wistar rats were randomized into two groups: the control group (C, n= 8 animals) that received standard diet/water and the obese group (OB, n= 8 animals) that were fed a high sugar-fat diet and water plus 25% of sucrose for 30 weeks. Nutritional analysis: body weight, adiposity index, food, water, and caloric intake. Obesity-related disorders analysis: plasma glucose, uric acid and triglycerides, HOMA-IR, systolic blood pressure, TNF-α in adipose tissue. Cardiac analysis included: TLR-4 and NF-ĸB protein expression, TNF-α and IL-6 levels. Comparison by unpaired Student’s t-test or Mann- Whitney test with a p-value < 0.05 as statistically significant.

**Results:**

The OB group showed obesity, high glucose, triglycerides, uric acid, HOMA, systolic blood pressure, and TNF-α in adipose tissue. OB group presented cardiac remodeling and diastolic dysfunction. TLR-4 and NF-ĸB expression and cytokine levels were higher in OB.

**Conclusion:**

Our findings conclude that, in an obesogenic condition, the inflammation derived from cardiac TLR-4 activation can be a mechanism able to lead to remodeling and cardiac dysfunction.

## Introduction

Obesity, defined as an excessive body fat accumulation that may impair an individual’s health,^1^is currently considered the most important nutritional disorder in both developed and underdeveloped countries.^2^Estimates show that in 2025 at least 18% of the adult population will be obese, a worrying condition since it can lead to several comorbidities, including cardiovascular diseases.^3 , 4^

One of the main causes responsible for the obesity epidemic is the excessive consumption of hypercaloric diets, which are rich in saturated fat and refined sugars, associated with a sedentary health style.^5 , 6^This modern lifestyle leads to adipose tissue hypertrophy that triggers, initially, a local inflammatory process, which may later affect and compromise the function of other organs, such as the heart.^7^This inflammation of metabolic origin results in the synthesis of the proinflammatory cytokines led by different pathways, among them: adipose tissue lipolysis,^8^adipocytes hypertrophy,^9^fatty acids from the diet,^10^and intestinal lipopolysaccharides (LPS) due to dysbiosis.^11^Within this context, free fatty acids and LPS are the main components involved in the inflammatory response, acting as Damage-Associated Molecular Patterns (DAMPs) and Pathogen-associated Molecular Patterns (PAMPs), respectively, which are recognized by some receptors, especially the Toll-Like Receptor 4 (TLR-4).^12^

TLR-4 belongs to a family of receptors usually expressed in cells of the innate immune system, such as macrophages, neutrophils, and lymphocytes, and exerts an important role to detect and recognize pathogens leading to the innate^13^and acquired^14^immune response activation. This receptor is responsible for the activation of the nuclear factor kappa B (NF-ĸB) with consequent synthesis of proinflammatory cytokines involved in the pathophysiology of several diseases, including heart diseases.^15^

However, the literature reports that the TLR-4 receptor is not expressed only in immune system cells. Cardiomyocytes also express this receptor and its activation, especially against infectious cardiac pathogens, contributes to the myocardial dysfunction process.^16^Although the involvement of the immune system in the development of cardiac pathologic hypertrophy is well established,^14 , 19 , 20^the TLR-4 pathway participation in the development and progression of obesity-related cardiac remodeling has not yet been clarified.^21^Considering the lack of studies regarding this topic, the aim of this study was to test the hypothesis that the TLR-4 receptor activation participates in the obesity cardiomyopathy process, due to production of cytokine through the NF-ĸB activation.

## Material and Methods

### Experimental Protocol

Male Wistar rats (n=16), 21 days of age, were obtained from *Universidade Estadual Paulista* (UNESP). Sample size was calculated by using SigmaStat for Windows, version 3.5 (Systat Software Inc., San Jose, CA, USA), considering the expected difference mean of 2.0, expected standard deviation of 1.0, test power of 90%, and α of 0.05. This calculation considered the adiposity index of previous studies published by our group.^22^The animals were randomly divided into two experimental groups: the control group (C, n= 8 animals) that received standard diet/water and the obese group (OB, n= 8 animals) that received high sugar-fat (HSF) diet and water plus 25% of sucrose for 30 weeks. The diet model is a well-established model to induce obesity previously published by our research group.^25^The HSF diet contained soybean meal, sorghum, soybean peel, dextrin, sucrose, fructose, lard, vitamins, and minerals, plus 25% sucrose in drinking water. The control diet contained soybean meal, sorghum, soybean peel, dextrin, soy oil, vitamins, and minerals.

Food and water were offered *ad libitum* and the animals were kept in individual cages, in an environment with controlled temperature (24 ± 2°C), humidity (55 ± 5%), and light-dark cycle (12-12h). The study protocol (CEUA 1265/2018) was approved by the Ethics Committee on Animal Experimentation of the Botucatu School of Medicine, UNESP, São Paulo, Brazil, and followed the recommendations from the Guide for the Care and Use of Experimental Animals *.* ^26^At the end of 30 weeks, the animals were fasted for 8h, then euthanized and the material was collected for analysis.

### Nutritional Evaluation

Food and water consumption were evaluated daily. Caloric intake was determined by multiplying the energy value of each diet (g × Kcal) by daily food intake. For the OB group, caloric intake also included water calories (0.25 × 4 × mL consumed). The animals’ body weight was measured weekly. After euthanasia, visceral fat (VAT), epididymal fat (EAT), and retroperitoneal fat (RAT) were collected and used to calculate the adiposity index (AI) by the formula: [(VAT + EAT + RAT) / ANIMAL WEIGHT] x100.

### Obesity-related disorders analysis

Plasma glucose and triglycerides, (BioClin, Quibasa Química Básica Ltda., Belo Horizonte, Minas Gerais, Brazil) were measured by colorimetric-enzymatic method in an automatic enzymatic analyzer system (Chemistry Analyzer BS-200, MindrayMedical International Limited, Shenzhen, China). Insulin (EMD Millipore Corporation, Billerica, MA, USA) was analyzed in plasma by ELISA using a commercial kit. The reading was performed by Spectra Max 190 microplate spectrophotometer (Molecular Devices®, Sunnyvale, CA, USA).

The Homeostatic Model Assessment ** (HOMA-IR), which allows evaluating insulin resistance, was also calculated according to the following formula: fasting insulin (μUI / mL) x fasting glucose (mmol / L) / 22.5.^27 , 28^

The Systolic blood pressure (SBP) evaluation was assessed in conscious rats by the non-invasive tail-cuff method with a NarcoBioSystems® Electro-Sphygmomanometer (International Biomedical, Austin, TX, USA). The animals were warmed in a wooden box (50 x 40 cm) between 38–40°C with heat generated by two incandescent lamps for 4–5 minutes to stimulate arterial vasodilation. After this procedure, a cuff with a pneumatic pulse sensor was attached to the tail of each animal. The cuff was inflated to a pressure of 200 mmHg and subsequently deflated. Blood pressure values were recorded on a Gould RS 3200 polygraph (Gould Instrumental Valley View, Ohio, USA). The mean of three pressure readings was recorded for each animal.

Since obesity is associated with an inflammatory condition in adipose tissue, TNF-α and IL-6 levels were evaluated. Epididymal adipose tissue (400 mg) was triturated with 2 ml of phosphate buffer saline (PBS) (pH 7.4) and then centrifuged at 3,000 rpm and 4°C for 10 min. Using the supernatants, TNF-α and IL-6 were measured using commercial ELISA kits (R&D Systems) according to the manufacturer’s instructions. The values were normalized by the protein amounts of each sample quantified by the Bradford method^29^and the results are expressed in picogram/g protein (pg/g protein). Epididymal adipose tissue was selected for presenting a similar inflammation pattern to that found in visceral fat.^30^

### Echocardiographic Analysis

The analysis was performed on live animals by transthoracic echocardiography, with a Vivid S6 system equipped with a multifrequency ultrasonic transducer of 5.0 to 11.5 MHz (General Electric Medical Systems, Tirat Carmel, Israel). The animals were lightly anesthetized by intraperitoneal injection with a mixture of ketamine (50 mg/kg) and xylazine (1 mg/kg) and placed in a left decubitus position. The structural measurements of cardiac images were obtained in the one-dimensional mode (M-mode) guided by the images in the two-dimensional mode with the transducer in the parasternal position, minor axis. Left ventricular (LV) evaluation was performed with the M-mode cursor just below the mitral valve plane at the level of the papillary muscles. All examinations were performed by the same examiner and obtained according to the main method recommended by the American Society of Echocardiography. The aorta and left atrium images were obtained by positioning the M-mode cursor to plan the aortic valve level. The following cardiac structures were evaluated: left ventricular diastolic diameter (LVDD); left ventricular posterior wall thickness at end-systole (LVPWS); Left ventricular systolic interventricular septum thickness (LVSIS); aorta diameter (AD); left atrium (LA); and the relative wall thickness (RWT) of the LV. The LV systolic function was evaluated by cardiac output and also by heart rate (HR), since it is a cardiac systolic function modulator. The LV diastolic function was assessed by the transmitral flow early peak velocity (E); deceleration time of E wave (Dec. time). The study was supplemented by evaluation by early (E’) and late (A’) diastolic tissue Doppler of the mitral annulus (arithmetic mean travel speeds of lateral and septal walls), and the wave ratio (E/E’ and E’/A’).

### Cardiac tissue analysis

#### Inflammation

One hundred milligrams (100mg) were homogenized in PBS and the supernatant was used. TNF-α and IL-6 quantification were performed by ELISA using a commercial kit (Linco Research Inc., R & D Systems, Millipore and B-Brigde International Inc.). The reading was performed by Spectra Max 190 microplate spectrophotometer (Molecular Devices®, Sunnyvale, CA, USA). The results were corrected by the total protein amount according to the Bradford method.

## Real time PCR

The frozen left ventricular fragment was homogenized in TRIzol® for extraction of ribonucleic acid (RNA). Afterward, the RNA was subjected to reverse transcription, conversion of RNA to complementary deoxyribonucleic acid (cDNA), by the action of the enzyme reverse transcriptase, using the SuperScript II First-Strand Synthesis System for RT-PCR® Invitrogen, São Paulo, Brazil. The obtained cDNA was used in the polymerase chain reaction (PCR) using ready-made assays (Applied Biosystems, CA, USA) containing TaqMan MGB (FAM) primer and primer specific for TLR-4 (Rn00569848_m1).

## Western Blot

The heart fragments were homogenized in lysis buffer and centrifuged. The supernatant was collected and the protein concentration was analyzed by the Bradford method.^29^After quantification, the cardiac protein extracts were diluted in a buffer solution containing 50 mM Tris-HCl (pH 6.8), 200 mM 2-Mercaptoethanol, 2% Sodium Dodecyl Sulfate (SDS), 0.1% bromophenol blue, and 10% glycerol. The dilutions (50ug) were heated and subjected to sodium dodecyl sulfate/polyacrylamide gel electrophoresis (SDS-PAGE) in 10% polyacrylamide gels. After electrophoresis, the proteins were electrotransferred to nitrocellulose membranes (Bio-Rad Biosciences; NJ, USA). Non-specific binding sites of the primary antibody to the membrane were blocked by incubation with 0.5% skimmed-milk powder solution, dissolved in TBS-T buffer pH 7.4. The membrane was then washed three times in basal solution and incubated overnight with TLR-4 specific primary antibody (sc293072), total NF-ĸB (sc8008), phosphorylated NF-ĸB (ser536) (sc33020). β- actin was used as an internal control (sc47778). After incubation, the membranes were washed and incubated with the respective secondary antibodies. Finally, immunodetection was performed by the chemiluminescence method, according to the manufacturer’s instructions (ECL SuperSignal® West Pico Chemiluminescent Substrate – Thermo Scientific, Rockford, IL, USA, 34080), and analyzed by means of a densitometer (GS-710 calibrated imaging densitometer, Bio-Rad lab, CA, USA).

## Statistical analysis

The data were submitted to Kolmogorov-Smirnov normality test. Parametric variables were compared by unpaired Student’s *t* -test and the results are expressed as mean ± standard deviation. Non-parametric variables were compared by Mann- Whitney test and the results are expressed as median (interquartile range 25-75). Statistical analyses were performed using Sigma Stat for Windows, version 3.5 (Systat Software Inc., San Jose, CA, USA). A p-value < 0.05 was considered statistically significant.

## Results

Table 1 shows the nutritional parameters. OB animals consumed less food, but more water compared to the control group, reflecting a similar caloric intake. The OB group also presented obesity characterized by increased body weight and adiposity index.

**Table 1:** – Nutritional parameters

Characteristics	Groups						
	Control (n=8)			OB (n=8)			p value
**Final body weight (g)**	493	±	50.8	583	±	75.9	0.001*
**Adiposity index (%)**	4.79	±	0.73	8.68	±	1.76	0.0004*
**Food consumption (g/day)**	23.92	±	2.37	13.0	±	2.20	0.0001*
**Water consumption (ml/day)**	34.6	±	5.08	41.6	±	3.47	0.001*
**Caloric intake (Kcal/day)**	85.9	±	8.52	98.3	±	8.35	0.07

OB: obese group. Data expressed as mean ± standard deviation. Comparison by unpaired Student’s t-test. *indicates p < 0.05.

Regarding the obesity-related disorders, the OB group presented higher glucose, triglycerides, and uric acid, insulin resistance, increased systolic blood pressure, and adipose tissue inflammation, with elevated TNF-α and IL-6 levels compared to the control group ( Table 2 ).

**Table 2:** – Obesity-related disorders

Characteristics	GROUPS						
	Control (n=8)			OB (n=8)			p-value
Glucose (mg/dL)	75.7	±	2.02	105	±	17.9	0.02*
Triglycerides (mg/dL)	63.5	±	18.6	104	±	25.4	0.003*
Uric acid (mg/dL)	0.44	±	0.09	0.62	±	0.20	0.04*
HOMA-IR	5.90	±	2.32	30.9	±	17.0	0.004*
Systolic Blood Pressure (mmHg)	121	±	5.77	128	±	6.48	0.03*
TNF-α Adipose tissue (pg/g protein)	52.7 (46.6 – 62.5)			152 (117 – 219)			0.001*
IL-6 Adipose tissue (pg/g protein)	13.0 ± 9.2			94.5 ± 33.3			< 0.001*

OB: obese group; HOMA-IR: Homeostatic Model Assessment. Data expressed as mean ± standard deviation or median (interquartile range). Comparison by unpaired Student’s t-test or Mann-Whitney. * indicates p < 0.05.

The echocardiographic analysis is presented in Table 3 . At the end of 30 weeks, the OB group showed cardiac remodeling, characterized by reduced LVDD and increased LVPWS, LVSIS, LA, and AD. Moreover, OB animals presented diastolic dysfunction, represented by changes in the E wave, E wave deceleration time, and E’/A’.

**Table 3:** – Echocardiographic analysis

Characteristics	GROUPS						
	Control			OB			p-value
**LVDD (mm)**	7.20	±	0.20	6.70	±	0.56	0.031*
**LVDD/BW**	14.7	±	1.6	11.9	±	1.3	0.002*
**LVPWS (mm)**	3.03	±	0.31	3.35	±	0.21	0.033*
**LVSIS (mm)**	3.35	±	0.21	3.57	±	0.19	0.045*
**RWT**	0.45	±	0.05	0.51	±	0.09	0.098
**AD (mm)**	3.73	±	0.12	3.99	±	0.13	0.001*
**LA (mm)**	4.75	±	0.12	5.17	±	0.38	0.011*
**HR (bpm)**	234	±	39	295	±	26	0.002*
**Cardiac Output**	0.91	±	0.01	0.88	±	0.06	0.142
**Deceleration time (ms)**	47.2	±	3.0	50.6	±	2.7	0.035*
**E wave**	68.3	±	5.2	73.1	±	3.3	0.046*
**A wave**	40.7	±	3.7	45.7	±	6.1	0.062
**E/A ratio**	1.68	±	0.08	1.61	±	0.016	0.327
**E'/A' medium ratio**	1.53	±	0.14	1.26	±	0.25	0.018*
**E/ E’ medium ratio**	12	±	1.3	13.5	±	1.86	0.094

OB: obese group; LVDD: left ventricular diastolic dysfunction; BW: body weight; LVPWS: left ventricular posterior wall thickness at end-systole; LVSIS: left ventricular systolic interventricular septum thickness; RWT: relative wall thickness; AD: aorta diameter; LA: left atrium; HR: heart rate. Transmitral flow early peak velocity (E wave); deceleration time: deceleration time of the E wave. Doppler early (E’) and late (A’) diastolic of the mitral annulus (arithmetic average travel speeds of lateral and septal walls), and the ratio (E/E’ and E’/A’). Data expressed as mean ± standard deviation. Comparison by unpaired Student’s t-test. * indicates p < 0.05.

Regarding the TLR-4 gene and protein expression in cardiac tissue, it is possible to verify that both were increased in the OB group ( Figure 1 ).

**Figure 1:** – TLR-4 expression in cardiac tissue. (A) Gene expression by real-time PCR; (B) Protein expression by Western Blot. Results expressed as mean ± standard deviation. Comparison by unpaired Student’s t-test. * indicates p < 0.05; n = 8 animals/group.

NF-ĸB phosphorylation in cardiac tissue was also higher in the OB group ( Figure 2 ), resulting in increased cytokines, since this group showed higher TNF-α and IL-6 levels compared to the C group ( Figure 3 ).

**Figure 2:** – Phospho and total NF-ĸB protein expression in cardiac tissue. Results expressed as mean ± standard deviation. Comparison by unpaired Student’s t-test. *indicates p < 0.05; n = 8 animals/group.

**Figure 3:** – Cardiac cytokines in cardiac tissue. (A) IL-6 (pg protein/g); (B) TNF-α. Results expressed as median and interquartile range. Comparison by Mann-Whitney test. * indicates p < 0.05; n = 8 animals/group.

## Discussion

This study hypothesized that the TLR-4 receptor activation participates in obesity-related cardiac disease by triggering cytokine production via NF-ĸB. In order to induce obesity and disorders related to excessive body fat, the animals in the OB group were submitted to a HSF diet for 30 weeks. At the end of the experimental period, the results show that these animals presented higher adiposity index and several disorders, such as hyperglycemia, increased uric acid, insulin resistance, hypertriglyceridemia, elevated SBP, increased TNF-α and IL-6 levels in both heart and adipose tissue, confirming the efficacy of the diet model used.^22^

The coexistence of obesity-related disorders — such as insulin resistance, diabetes, and dyslipidemia — associated with adipose tissue dysfunction, characterized by adipokine imbalance, promote maladaptive responses in the heart, such as myocyte hypertrophy, contractile dysfunction, and cardiac remodeling, which contribute to both the development and progression of chronic heart failure.^31^This condition was confirmed in this study, since the echocardiogram evaluation showed cardiac remodeling and diastolic dysfunction in the OB group. It is important to emphasize that the concentric characteristics of cardiac remodeling observed in the OB group are the result of an increased functional overload and the maintenance of this condition, leading to cardiac diastolic dysfunction. Similar results have been related to the diet-induced obesity model in the literature.^34^

The literature reports that cardiac remodeling can also be led by high concentrations of proinflammatory cytokines, such as TNF-α and IL-6.^35 , 36^TNF-α has been implicated in the development of left ventricular dysfunction, left ventricular remodeling, increased cardiac myocyte apoptosis and its direct action is exerted by TNF-α receptors, which are expressed by almost all nucleated cells. Increased IL-6 levels can also induce myocyte hypertrophy and myocardial dysfunction.^37^It is important to emphasize that these cytokines can be produced in response to TLR-4 pathway activation, and also L-6 seems to be released in direct response to TNF-α,^35^exacerbating the cardiac changes due to inflammatory condition. Therefore, our findings confirm the evidence regarding obesity cardiopathy and inflammation, since the cardiac levels of TNF-α and IL-6 were higher in the OB group.^18 , 37^

The involvement between proinflammatory cytokines and obesity-related cardiac remodeling induced by diet can be attributed to several causes.^18 , 38^Thus, the aim of this study was to evaluate cardiac TLR-4 activation as responsible for triggering the inflammatory process. It was observed that the OB group presented higher TLR-4 gene and protein expression together with increased NF-ĸB phosphorylation, confirming the activation of this pathway as a mediator of inflammation. The literature reports that this receptor can be activated by LPS of gram-negative bacteria, but also by fatty acids.^39^In obesity, the adipose tissue lipolysis represents an important source of free fatty acids, capable of activating the inflammatory pathway.^40 , 41^This catabolic process can occur due to insulin resistance, once the adipose tissue becomes resistant to the hormone antilipolytic effect. Together with insulin resistance, increased TNF-α and IL-6 levels are also able to induce lipolysis in adipose tissue.^42 , 43^These two lipolytic conditions were presented by the OB group. Moreover, associated with these mechanisms described above, some researchers report that the Western diet pattern is associated with changes in the intestinal microbiota, making this organ more permeable, allowing the translocation of pathogenic bacteria to the circulation.^42^Bacterial LPS can be recognized by TLR-4, triggering the NF-ĸB activation with consequent cytokine synthesis. Although this mechanism was not evaluated in this experiment, this relationship is already well established.^43^In addition, the diet composition used in this study is also directly related to the TLR-4 receptor activation, since the saturated fatty acids offered in the diet have similar structures to bacterial LPS, being also able to be recognized, leading to an inflammatory process via TLR-4 pathway.^46 , 47^

## Conclusion

In summary, all these mechanisms may have been activated in a synergistic way, enhancing the production of cytokines that play a fundamental role in the development of cardiomyopathies. Thus, this study shows that the innate immune response through TLR-4 receptor activation is one of the mechanisms that can contribute to the onset of the myocardial inflammatory process in obesity. Therefore, since no studies were found in the literature showing an interaction between this inflammatory pathway, heart disease and obesity, our findings conclude that in an obesogenic condition, the inflammation derivative from cardiac TLR-4 activation is a new mechanism which can lead to remodeling and cardiac dysfunction.
